# The Clinical Significance of the Diseases of Children

**Published:** 1898-02

**Authors:** H. W. Davis

**Affiliations:** St. Paul


### Selections and Abstracts.

### THE CLINICAL SIGNIFICANCE OF THE DISEASES OF CHILDREN.

By H. W. DAVIS, M. D., St. Paul.

Read before the Ramsey County Medical Society, November 29, 1897.

On a physician's ability to manage children oftentimes depends not only his pecuniary success, but also the health and life of the children. How comparatively easy to diagnosticate a simple ailment in an adult, but oftentimes how difficult in a restless, frightened, crying child. In speaking of a child, I mean those under seven or eight years of age. Those older than that are as able to give as lucid a description of their feelings as many older ones, but before the age of seven the symptoms are nearly all objective, and it is only by close observation that we arrive at a correct diagnosis.

The physician must be one who is endowed with a large share of the milk of human kindness. One who is impatient or in a hurry will seldom arrive at an intelligent idea of the trouble. Who can not recall visiting a child which immediately began to cry when the doctor was ushered in, and kept on crying until in disgust a prescription was written and an exit hastily made, only to find in the morning a very ill little patient, docile enough now in a semi-comatose condition, the prescription having done no good, and possibly harm, because enough forbearance was not practiced, and the confidence of the child was not gained, nor a correct diagnosis made. Therefore, plenty of time should be taken to get acquainted, during which time the hands, if cold, should be warmed thoroughly, as nothing startles a child more than the contact of cold hands.

A complete history of the illness should be gotten. This is often impossible, for so little attention is paid to the daily care of most children that, unless they are infants in arms, no knowledge is gained of their diet or alvine dejections. The first stage of meningitis is often only recognized by the completeness of the history, the existing irritability of the stomach being only a result of that disease. So also in the exanthemata. A few days of indisposition may be the prodromal symptoms of these diseases, and are considered of so little importance by the mother or nurse that the information is only elicited by careful questioning. If the disease is of the respiratory organs, the character of the cough should be sought; if of the digestive organs, the minutest details of feeding should be inquired into. The soiled napkins should be inspected, for the word of the attendant will often lead us into error. To illustrate: A child was persistently passing a great many curds in spite of a carefully selected diet, or thought to be so. The food was modified and changed several times. No soiled napkins had been available when the physician called, until finally, the trouble persisting, he insisted upon examining a napkin, and found that comparatively rare condition, a fatty diarrhœa; the curds instead of being casein were fat. This is a personal experience.

The growth and development of the child are important. Also its weight, process of dentition, when it first walked, exposure to contagious diseases; also the character of the labor, whether easy or difficult. Heredity is important in chronic disease, and should be carefully examined. I think I am safe in saying that nine-tenths of the diseases of children are of the respiratory and digestive organs. So special attention is always directed to these affections, not overlooking the existence of a prevailing epidemic.

Children also, on account of the susceptibility of their nervous systems, show grave symptoms from slight functional causes, and when apparently desperately ill recover in a few hours or days. Or the symptoms may be so obscure that a diagnosis is not made during life, and even an autopsy fails to clear the atmosphere of doubt. As Holt says in his excellent book on children, "What is gained by inspection depends almost entirely upon the powers of observation of the physician." One who has no difficulty in arriving at a diagnosis in adults by a system of questions and answers is at a loss in children, but with patience and observation it becomes almost as easy.

When visiting a child with an acute disease several points are to be noted. If the child is asleep, the posture, if it lies on the side, back or face, if the legs are drawn up or the head retracted, if the sleep is quiet, or does it cry out while sleeping? Does it keep involuntarily swallowing, indicating a sore throat? Do the eyelids twitch, which may be the warning of an approaching convulsion? Do the features contract from time to time, indicating pain? The respiration will be especially noted. Is it rapid and shallow and irregular, or quiet and even and slow? Is there that peculiar little catch to it, which indicates pain, as in pleurisy or pneumonia? Is there any cyanosis, as if some obstruction existed? Is it noisy, as in croup? Does the child breathe through the mouth, as when adenoids, tonsillitis or pharyngitis exists? Is there any reces- on of the supraclavicular or suprasternal regions?

The pulse, its volume and rhythm, its rapidity is not of so much importance. A slow irregular pulse is almost always indicative of meningitis, a rapid irregular one is not necessarily of serious import.

The conditon of the skin is noted. Is it moist or dry? Are the extremities cold or warm? Is there any cyanosis? Are the fingers clubbed, as in tubercular tendencies? Are there any eruptions? And for this purpose the clothing should be removed, as it should be in all careful examinations. The diaper can usually remain on. Oftentimes the only eruption of scarlet fever will be noted on some part of the body covered by the clothing. The degree of emaciation or plumpness can now be noted.

As the cry of the child is its only means of expression, due attention and study should be given to it, as its character is of undoubted assistance as an aid to diagnosis. The cry is strung to a great many different tunes, and each one is significant. The cry of hunger is a short, heart-rending one, which is relieved when anything is inserted into the mouth, and ceases when hunger is appeased. When crying from hunger there will be intervals in which the fingers are loudly sucked. The cry of indigestion resembles that of hunger, but is renewed soon after the hunger has been apparently relieved. The cry of intestinal pain is sharp and piercing, and ends gradually in sobbing, and the child goes to sleep, only to start up suddenly and repeat it, the legs being sharply flexed. In earache, the common disorder of infants, the cry is of the same character, and can only be differentiated by the existence of other symptoms. Then there is the suppressed cry of pleurisy or pneumonia, accompanied by the catchy respiration. I have noticed another peculiarity in children in pneumonia, or in pleurisy, that the pain is often referred to the abdomen; if on the right side it resembles that of appendicitis, and is quite puzzling in the absence of a cough. The cry of weakness is a continous whine which lasts during most of the waking hours. There is the cry of temper and habit. The former seldom occurs before the sixth or seventh month. It is accompanied by extension of the extremities and retention of the breath, and often frightens the young mother on account of the cyanosis that occurs. The cry of habit is the hardest to estimate. It comes from over-indulgence. The child cries to be held, to be carried, when it is wet to be put down, and from every imaginable cause. The babies in the Babies' Home invariably cry for several days after their admission, but if they receive attention at stated intervals they soon cease crying, but, on the other hand, if they are taken up every time they cry, they keep it up indefinitely. So the only way to do is to let them cry until they are exhausted for several times, and then it ceases.

The condition of the pupils and lids is important. If there is intolerance to light without elevation of temperature or variation of pulse, a simple headache exists. If with these symptoms and added to them, a dilatation of the pupils, the meninges are affected. In all febrile conditions of children the pupils are usually dilated. It should be noted if the pupils react to light. Also the presence of corneal ulcers and interstitial keratitis, as is so common in hereditary syphilis. The glands at the side of the neck are factors to the diagnosis. They may indicate diphtheria, scarlet fever or simple adenitis, or, if several are inflamed and the others enlarged, probably they are tubercular. In diphtheria and scarlet fever usually the deep cervical are affected. In simple adenitis the superficial, cervical or submaxillary are the ones.

The presence of nasal discharge indicates either influenza, diphtheria or scarlet fever, or if it is chronic, syphilis; bloody mucus or pus from the nose is usually from diphtheria. In a large proportion of cases of diseases of children, the history of the illness and intelligent observation by the physician will lead him to form a correct diagnosis, but a physical examination should not be omitted.

In regard to the temperature, I do not depend upon the sense of touch, but use a thermometer in the rectum to the age of five years, when they can hold it safely in the mouth. The heat regulation center in childhood acts so imperfectly that the temperature is affected by trivial causes. By personal observation in most children the daily range is from 98 degrees to 99-5 degrees, so unless the temperature is continuously elevated for a considerable time, it does not indicate anything. A simple rise occurs and in a few hours it is normal. In an illness lasting several days, or a wasting disease, when possibly the temperature is subnormal, the daily use of the thermometer is needful.

The experimenter has succeeded in raising infants' temperature one to five degrees by the use of artificial heat, hot bottles applied, and I have seen the hot air from a register raise the rectal temperature two degrees. These phenomena are hard to understand.

The examination of the urine should not be neglected. Diabetes is a rare disease in children, but it should not be forgotten that it may occur. I have never seen but two cases occurring in children under ten years of age, but in both it accompanied tubercular meningitis. I think a urinalysis should be made after all exanthemata and diphtheria. Many of those cases that are so long in convalescing are due to albuminuria, and when I speak of the exanthemata, I mean not only scarlatina but varicella, rubella and rubeola, and especially the latter. Measles should receive more careful consideration than is usually accorded it. The sequelæ are nearly as numerous and as severe as those that follow scarlet fever. In New York City last year more children under two years of age died of measles than from any other disease except intestinal troubles. I believe the disease should be quarantined.

Under the head of albumen in the urine I desire to relate a peculiar condition that came under my notice. A child four years old was recovering from an exhausting disease, and owing to some transient stomach trouble, nutrient enemas were given. These were composed of milk, egg and beef peptonoids. After the first enema I had cause to examine the urine and found an abundance of albumen, much to my surprise, as none has existed before. This was in the afternoon, the injection having been given at noon. The next morning, no albumen existed. Gave another nutrient enema and the albumen reappeared four hours afterward, tried it the following day with the same result. No more injections were given and there has been no more albuminuria. I do not pretend to explain why the albumen should so quickly appear in the urine after being injected into the bowels, but that it did so is unmistakable; evidently no nephritis existed.

By examination of the napkin one may determine if crystalline uric acid is deposited, or if the urine is highly concentrated. In male infants the urine may be gathered by a condom, and in females by a small dish placed under the diaper, and the further elucidation of urinalysis may throw more light on children's diseases, especially the gastro-intestinal variety.

The examination of the thorax will call forth all one's ingenuity, but with a little patience can be accomplished. Of course the clothing should be removed and the atmosphere of the room should be at least seventy-two degrees. The condition of the chest is noted, whether there are any deformities, as from rickets. If the expansion is symmetrical, or any bulging of the intercostal spaces. The chest wall is thinner than in an adult, owing to a lack of muscular development, and more elastic on account of the cartilaginous condition of the framework. For these reasons all pulmonary sounds are puerile or exaggerated. Also the bronchi are relatively larger. A slight bronchial rale may be felt by palpation.

Percussion should be practiced with warm hands, and done lightly. The percussion note is usually exaggerated, almost tympanitic, and in the intraclavicular region and between the scapulæ there may be the cracked-pot sound. Ausculation by the use of the ear applied to the chest is preferable to the use of the stethoscope, as it does not frighten the child as much, and if one is used to that method it is as efficient. The posterior part of the chest should be examined first, as usually the first signs of diseases are found there. Owing to the loud, almost bronchial respiration of children it will be necessary to compare that of both sides to avoid error. It should also be remembered that a child's breathing is often irregular, several long breaths, and then a few shallow, faint, quick ones. It is better that ausculation should be practiced before percussion, as it is very difficult to do when the child is crying, and upon it usually depends the diagnosis.

In differentiating a bronchial rale from a pleuritic friction sound, it is sometimes necessary to cause the child to cry and cough and expel the mucus. Areas of consolidation may exist without affecting the percussion note on account of the surrounding emphysematous condition. Flatness always indicates fluid, but when there is fluid usually you also get bronchial breathing, somewhat different from that of consolidation, not quite so distinct.

The apex beat of the heart is usually in the mammary line, or just outside. Heart murmurs are rarely heard before the age of two years unless they are congenital. If the spleen can be felt below the ribs, it is enlarged unless it is pushed down by a deformity of the chest. In acute diseases enlargement of the spleen means typhoid fever or tuberculosis. In chronic diseases, malaria, anæmia or syphilis. The liver is usually detected a finger's breadth below the ribs.

The condition of the abdomen should be noted; if it is retracted, as in meningitis, or if tympany exists, as in acute intestinal diseases or in rickets. Also determine if phimosis or balanitis is present. I have left the examination of the throat, as it seldom can be done without crying, and, therefore, should be deferred until the last in the examination, and then requires a good light and a quick glance, and often it is necessary to do it forcibly. Often the first signs of the eruption of measles or varicella are seen on the hard palate.

Redness of the fauces indicates either a simple pharyngitis or scarlet fever, although diphtheria may exist without a membrane.

Another important item in an examination is the rectum. It is very common for an ulcer or a fissure to exist. With a history of constipation, and especially if the child is restless and in pain after defæcation, the rectum should be examined. It often escapes the mother's notice, and she is surprised when it is pointed out. The insertion of the finger and scraping the ulcer with your finger nail is usually all that is needed to effect a cure.

In the examination of children, trifles should be considered. The relative age, weight, size of cranium, mentality, etc. No harsh measures should be used if they can be avoided, and by tact as satisfactory a diagnosis can be made as in adults.

I feel as though I ought to offer an apology for this rambling talk. Some of it I have read, and most of it I have jotted down from observation, but it is only by telling such clinical experiences that they are of any benefit to others, so I may be pardoned.—*Northwestern Lancet.*

